# Role of the vaginal microbiome in miscarriage: exploring the relationship

**DOI:** 10.3389/fcimb.2023.1232825

**Published:** 2023-09-13

**Authors:** Marwa Saadaoui, Parul Singh, Osman Ortashi, Souhaila Al Khodor

**Affiliations:** ^1^ Research Department, Sidra Medicine, Doha, Qatar; ^2^ College of Health and Life Sciences, Hamad Bin Khalifa University, Doha, Qatar; ^3^ Women’s Services Department, Sidra Medicine, Doha, Qatar

**Keywords:** pregnancy complications, pregnancy loss, vaginal microbiota, vaginal dysbiosis, inflammation

## Abstract

Miscarriage is a devastating pregnancy loss that affects many women worldwide. It is characterized as a spontaneous miscarriage that occurs before 20 weeks of gestation which affects more than 25% of pregnancies. While the causes of miscarriage are complex and multifactorial, recent research has suggested a potential role of the vaginal microbiota. The vaginal microbiome is a dynamic ecosystem of microbes that are essential for preserving vaginal health and avoiding infections. Vaginal dysbiosis has been accompanied with numerous adverse pregnancy complications, such as preterm birth. However, the effect of the vaginal microbiome in miscarriage is not fully understood. This review aims to investigate the link between vaginal microbiota and miscarriage. Also, we investigate the various mechanisms through which the vaginal microbiota may affect miscarriage. Additionally, we examine the implications of these research findings, specifically the possibility of vaginal microbiome screening and targeted interventions to prevent miscarriage.

## 1 Introduction

Miscarriage is a prevalent issue in obstetrics, affecting approximately 25% of pregnancies worldwide. Miscarriages can be divided into two categories based on time: early miscarriages, which occur before to 12 weeks of gestation, and late losses, which occurs between 12 and 22 weeks of pregnancy (Larsen et al., 2013; Al-Memar et al., 2020). Despite being common, the causes of the majority of miscarriages are still unknown (Larsen et al., 2013). Possible factors include uterine abnormalities (Chan et al., 2011), incorrect embryo selection (Kiecka et al., 2021), genetic (Demey et al., 1991; Branch et al., 2010) and epigenetic issues (Daher et al., 2012; Yin et al., 2012),, diseases of the embryo, immunological factors (Holers et al., 2002; Calleja-Agius et al., 2012),, endocrine variables (Cocksedge et al., 2009) chromosomal problems and lifestyle choices (Larsen et al., 2013) which may contribute to its occurrence.

During pregnancy, the composition of the vaginal microbiota has a substantial impact on the health of both the mother and the fetus, and the microbiome of pregnant women is characterized by greater stability and lower diversity than non-pregnant women (Freitas et al., 2017). Factors that can influence the vaginal microbiome include hormonal changes, food, sexual practices, medical treatments, and urogenital infections (Kroon et al., 2018; Noyes et al., 2018; Dall'Asta et al., 2021). Vaginal dysbiosis, which is a disruption of microbial balance in the vagina, is related with several pregnancy complications, including miscarriage and preterm birth (Brown et al., 2018; Juliana et al., 2020).

In a healthy pregnancy the vaginal bacterial composition remains stable, with a predominance of Lactobacillus species (Ravel et al., 2011; Aagaard et al., 2012; DiGiulio et al., 2015; MacIntyre et al., 2015; Gupta et al., 2020; Marangoni et al., 2021). According to research, there is a potential link between preterm delivery and a decrease in Lactobacillus species in the vaginal microbiota (Brown et al., 2018; Freitas et al., 2018; Al-Memar et al., 2020). However, further research is needed to completely understand the connection between miscarriage and the microbial composition of the uterus and vagina (Zhang et al., 2019; Al-Memar et al., 2020; Xu et al., 2020).

Research findings have confirmed that the composition of the vaginal microbiota may impact the likelihood of miscarriage. Dysbiosis can result in elevated inflammation and infections, these factors have been linked to an increase of the risk of miscarriage. Research has discovered that pregnant women with a well-balanced vaginal microbiota have a lower risk of experiencing a miscarriage compared to those with an imbalanced microbiome (Gunther et al., 2022) (Grewal et al., 2022). In recent years, there have been significant progresses in the study of the human microbiota and pregnancy (Vinturache et al., 2016; Gupta et al., 2020), the available literature on the correlation between the vaginal microbiome and miscarriage is currently sparse. Consequently, enhancing our comprehension of the association between these two factors may facilitate the identification of novel preventive measures and enhance pregnancy outcomes. The purpose of this review is to investigate how the vaginal microbiome affects the risk of miscarriage.

## 2 Miscarriage: types and symptoms

Around 23 million miscarriages happen global each year, resulting in an estimated average of 44 pregnancy losses every minute (Quenby et al., 2021). Research indicates that 10.8% of women who experience pregnancy loss have had one miscarriage, 1.9% have had two, and 0.7% have had three or more (Quenby et al., 2021). National registries and population-based cohort studies have shown a miscarriage rate ranging from 12.9% to 13.5% in some countries like Sweden, Finland, and Denmark (Hemminki and Forssas, 1999; Nybo Andersen et al., 2000; Adolfsson and Larsson, 2006). A prior Norwegian research that included all patients at a Oslo hospital from 2000 to 2002 estimated a 12% of them have miscarriage (Eskild et al., 2009).

Miscarriages can be divided into two categories based on time: early miscarriages, which occur before to 12 weeks of gestation, and late losses, which occurs between 12 and 22 weeks of pregnancy (Larsen et al., 2013; Al-Memar et al., 2020). Studies and nations may have different definitions of miscarriage and stillbirth, but in general, a stillbirth is defined as fetal death that happens at 20 weeks of gestation or later or includes a birthweight of 500g or more. Contrarily, miscarriages are often classified as births weighing less than 500g or fetal deaths happening before 20 weeks of gestation (Magnus et al., 2019). The presentation of miscarriage can vary from case to case. Vaginal bleeding is usually the primary indicator, which may be followed by cramping and pain in the lower abdomen. The passage of a blood clots or dark tissue, cramps and abdominal pain that are generally more severe than menstrual cramps, a low backache that ranges in intensity from mild to severe, and a decrease in pregnancy symptoms are all additional signs of a miscarriage. Miscarriage has a significant influence on both physical and psychological health, and research indicates that it can cause post-traumatic stress disorder and distress comparable to the death of a full-term baby. In the UK alone, an estimated 140,000 miscarriages occur every year, resulting in a cost to the economy of £471 million (Devall and Coomarasamy 2020). Despite its frequency, assessments to determine the cause of miscarriage are not common (Lathi et al., 2011).

Research associating variations in the vaginal microbiome with specific types of miscarriages is still in its preliminary stages. Nonetheless, there have been investigations into distinct categories of miscarriages with respect to their connection to the vaginal microbiome. These include:

1) Recurrent miscarriage (RM): Characterized by three or more consecutive miscarriages. RM affects around 1% of pregnancy (Garrido-Gimenez and Alijotas-Reig, 2015; Devall and Coomarasamy, 2020). Some studies propose that imbalances within the vaginal microbiota might contribute to the occurrence of recurrent miscarriages (Zhang et al., 2019; Grewal et al., 2022; Zhao et al., 2023).
2) A threatened miscarriage: This condition is characterized by vaginal bleeding and mild cramps, while the cervix remains closed. In half of cases, the bleeding stops, and the pregnancy continues normally. In the other half, the threatened miscarriage progresses to an inevitable miscarriage, resulting in pregnancy loss (Wahabi et al., 2011; Wahabi et al., 2018). Research suggests that dysbiosis in the vaginal microbiome increase the risk of threatened miscarriage (Chen et al., 2022).

## 3 Factors leading to miscarriage

**Figure 1:**
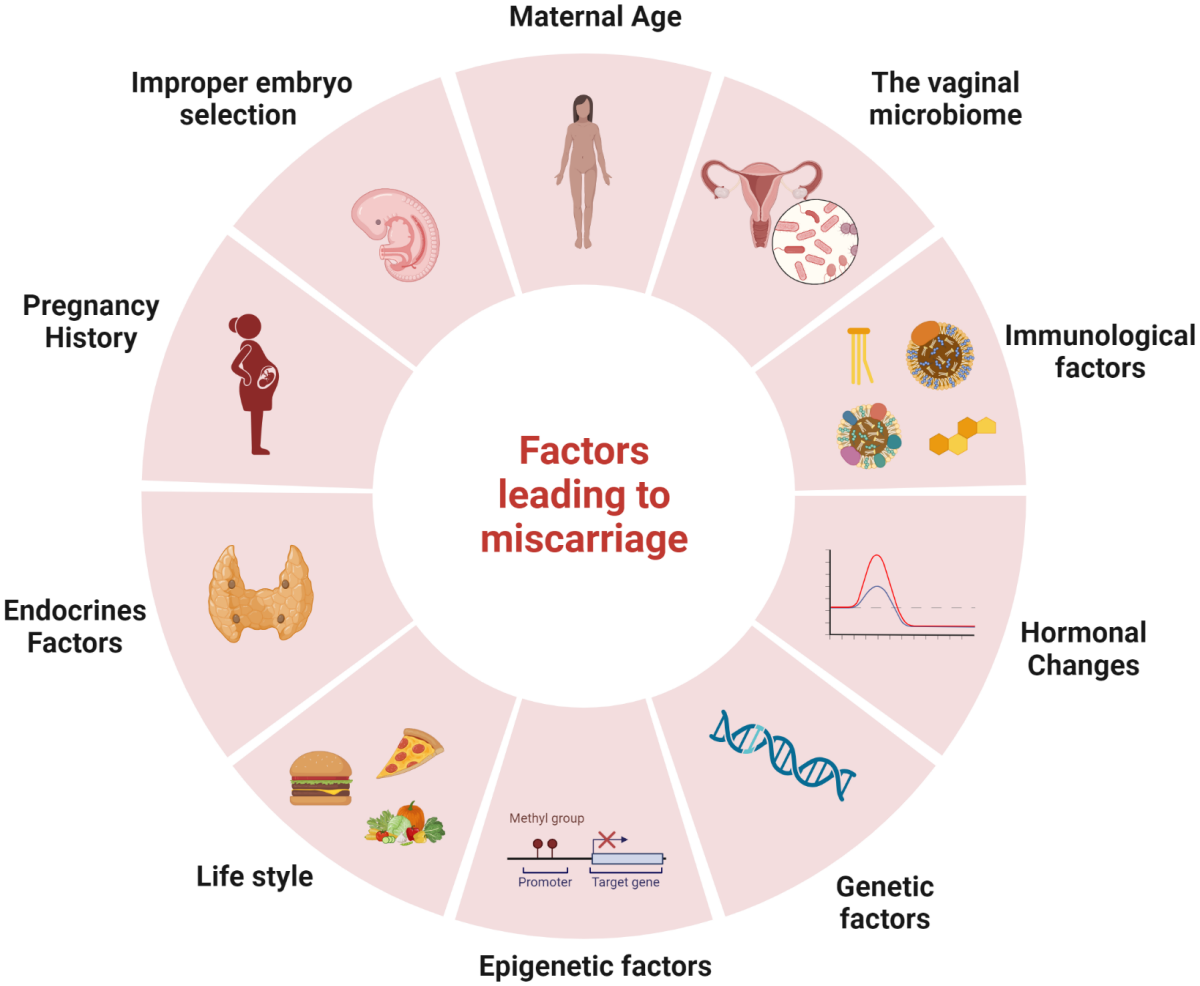
Factors leading to miscarriage: There are several factors that may increase the risk of miscarriage, including maternal age, the vaginal microbiome, immunological factors, hormonal changes, genetic factors, epigenetic factors, lifestyle, endocrines factors, pregnancy history and improper embryo selection. The figure was created with BioRender.com.

While the exact cause of most miscarriages remains unknown, various factors have been identified that may increase the risk of this unfortunate event (as illustrated in **Figure 1**). These factors include maternal age, genetics, hormones, immunology, and the environment (Agenor and Bhattacharya, 2015; Garrido-Gimenez and Alijotas-Reig, 2015),. Genetic factors, such as abnormal chromosomal rearrangements in the parents or abnormal genotypes or karyotypes in the embryo, may account for more than 50% of RM (Garrido-Gimenez and Alijotas-Reig, 2015). The age of the mother is the primary main risk associated with miscarriage, research shows that the probability of a pregnancy loss slightly increases in young mothers before significantly rising in older women (Nybo Andersen et al., 2000; de la Rochebrochard and Thonneau, 2002). Studies have consistently demonstrated that women over 30 years of age have a higher likelihood of miscarriage (de la Rochebrochard and Thonneau, 2002). Furthermore, over 50% of pregnancies at age 42 ended in a ectopic pregnancy, stillbirth or spontaneous abortion (Nybo Andersen et al., 2000) underscoring the significant and independent impact of maternal age on the probability of spontaneous abortion (Nybo Andersen et al., 2000; Nybo Andersen et al., 2000). This effect persists despite the strong correlation between maternal age, parity, and reproductive history (de la Rochebrochard and Thonneau, 2002). A Norwegian study found that women aged 25-29 had the lowest probability of miscarriage (9.8%), with the lowest risk occurring at 27 (9.5%), while the probability was the highest for women aged 45 and over (53.6%). For young pregnant women, under 20 years old, the risk was 15.8% (Magnus et al., 2019). This is consistent with previous research that have demonstrated an increased risk of miscarriage among women aged 35 and above, even after controlling for factors such as reproductive history and nationality (de la Rochebrochard and Thonneau, 2002).

Pregnancy history is another leading cause of miscarriage. A comparative study found that women who had experienced a previous pregnancy that ended in a stillbirth or miscarriage had a greater risk of pregnancy loss than women who had never been pregnant before (Magnus et al., 2019). Other outcomes in prior pregnancies, such as gestational diabetes and c-section delivery also increased the risk of pregnancy loss (Magnus et al., 2019).

Furthermore, there are significant differences across distinct phases of pregnancy and miscarriages. Particularly the first trimester is especially sensitive since most miscarriages occur during this time, often because of mutations in the developing fetus (Hyde and Schust, 2015) (Hardy and Hardy, 2015). While miscarriage risks generally decrease in the second trimester. While the risk of miscarriage generally diminishes in the second trimester, it remains pertinent to note that maternal health concerns or placental issues, for instance, can still underlie second-trimester miscarriages (Odendaal et al., 2019; Cheung et al., 2023). Miscarriages in the third trimester are rare but can be caused by various factors such as premature labor, infections, or placental problems (Giakoumelou et al., 2016).

According to research, different ethnic backgrounds may contribute to varying susceptibility to miscarriage (van der Hoorn and Lashley, 2022). Recently, it was discovered that Black ethnicity is linked to a higher incidence of miscarriage than White (Mukherjee et al., 2013; Quenby et al., 2021). Genetic traits inherent to specific ethnic groups may contribute to variations in immunological responses, hormone levels, and susceptibility to diseases. Additionally, cultural and socioeconomic factors could indirectly impact the likelihood of miscarriage by influencing healthcare accessibility and lifestyle choices (Yazdkhasti et al., 2015; Zheng et al., 2017).

## 4 Vaginal microbiome and miscarriage:

In contrast to other human microbiomes, the vaginal microbiota is relatively less diverse and characterized by a high prevalence of *Lactobacillus* species, particularly during pregnancy, as evidenced by various studies (Ravel et al., 2011; Gajer et al., 2012; Kervinen et al., 2019). The vaginal environment is a special place where the human immune system, vaginal epithelial cells, and microbes interact in complex ways (Ravel et al., 2011; Gajer et al., 2012). Although the bacterial communities in this ecosystem are composed of a variety of species, they can be grouped into 5 main community state types (CST) frequently observed in reproductive-age women of different ethnic and age groups: CST-I (dominated by L. crispatus), CST-II (dominated by *L. gasseri*), CST-III (dominated by *L. iners*), CST-IV (the pathogenic type, dominated by anaerobic bacteria), and CST-V (the stable type, dominated by *L. jensenii*) (Yamamoto et al., 2009; Ravel et al., 2011).

**Figure 2:**
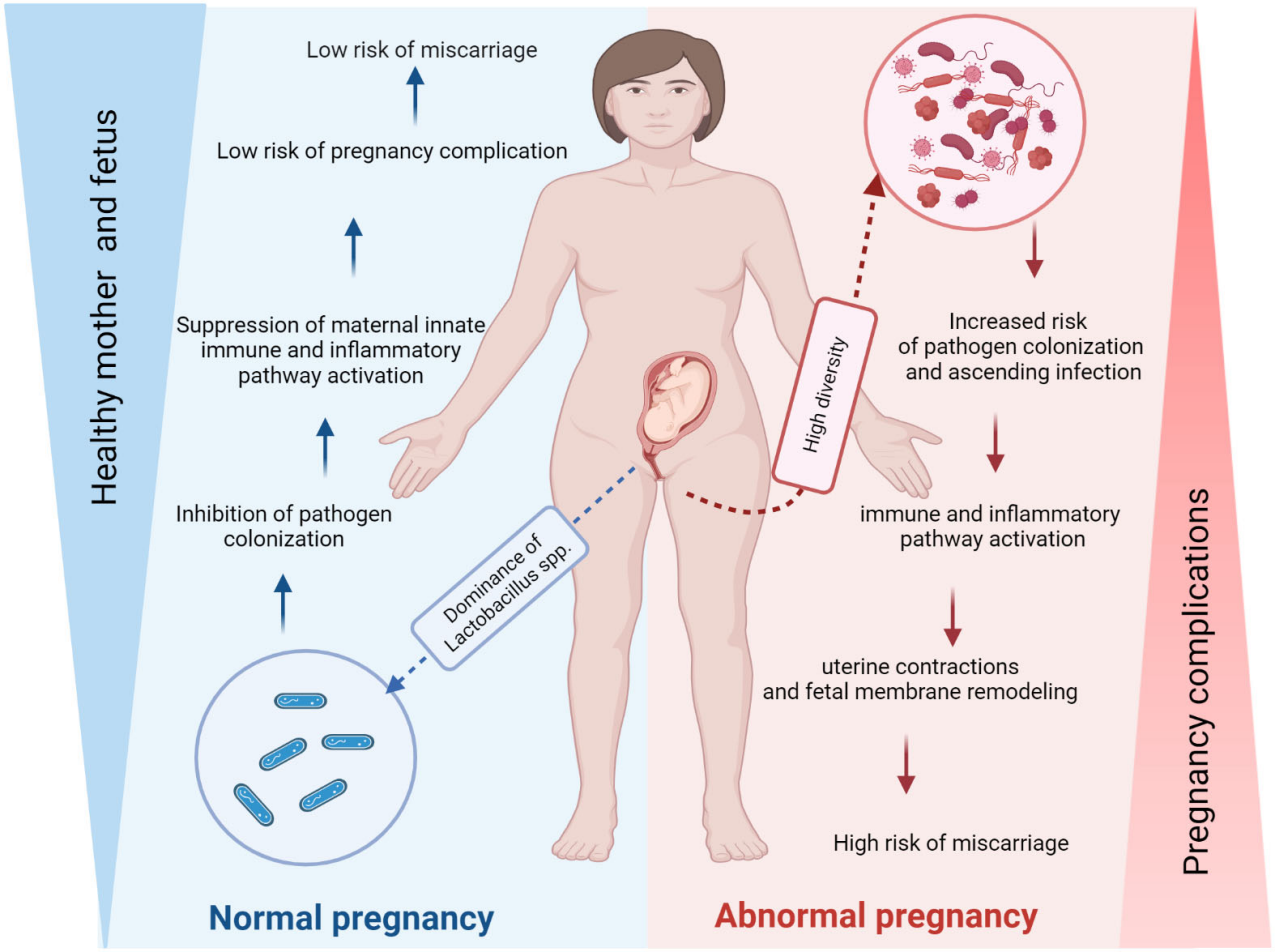
The vaginal microbiome may affect the risk of miscarriage. The composition of the vaginal microbiota plays a crucial role in conception and pregnancy. Indeed, the high diversity increased the risk of pathogen colonization and ascending infection that can activate the immune and inflammatory pathway, consequently, the uterine contractions and fetal membrane remodeling that increase the risk of miscarriage, in contrast, the dominance of Lactobacillus spp inhibit the pathogen colonization so the risk of pregnancy complication such as miscarriage is low (**Figure 2** **).** The figure was created with BioRender.com.

In a healthy state, the human vagina is primarily inhabited by *Lactobacillus* species, which play a crucial role in maintaining vaginal health. These beneficial bacteria help protect the vagina from antimicrobial substances and produce lactic acid to maintain a normal pH range of 3.6 to 4.5 (Boskey et al., 1999; O'Hanlon et al., 2013). However, the composition of the vaginal microbiota plays an important role in both conception and pregnancy (**Table 1**) (MacIntyre et al., 2015; Amabebe and Anumba, 2018). Indeed, the high diversity increased the risk of pathogen colonization and ascending infection that can activate the immune and inflammatory pathway, consequently, the uterine contractions and fetal membrane remodeling that improve the risk of miscarriage, in contrast, the dominance of *Lactobacillus spp* inhibit the pathogen colonization so low risk of pregnancy complication such as miscarriage (**Figure 2**). The composition of the vaginal microbiome can be influenced by various factors, including environmental conditions (such as antibiotics, contraception and pre- and probiotics), lifestyle (such as hygiene and sexual habits), individual characteristics (such as age, genetic and immunological factors, hormonal status, socioeconomic status and ethnicity), as well as general health (Macklaim et al., 2015; Brooks et al., 2017; Kervinen et al., 2019). Recent research has linked the vaginal microbiota diversity and richness to adverse pregnancy outcomes, such as preterm birth (Kumar et al., 2021) and miscarriage (Stout et al., 2017; Kervinen et al., 2019; Zhang et al., 2019). Essentially, numerous studies show a possible relationship between preterm delivery, a decrease in *Lactobacillus* spp. and an increase in bacterial biodiversity in the vagina (Brown et al., 2018; Freitas et al., 2018; Al-Memar et al., 2020). Despite advances in our understanding of the vaginal microbiome, we still know little about how its microbial composition directly affects miscarriage (Zhang et al., 2019; Al-Memar et al., 2020; Xu et al., 2020; Kiecka et al., 2021).

**Table 1:** Role of the vaginal microbiome in miscarriage.

The relationship between the vaginal microbiome and miscarriage	Reference
A meta-analysis was performed on various studies investigating the potential link between the vaginal microbiome and preterm birth, a known risk factor for miscarriage. The analysis revealed that preterm birth was more likely among women who had an abnormal vaginal microbiota. Furthermore, diver types of bacteria like *Gardnerella vaginalis* and *Ureaplasma* can increase the risk of pregnancy loss	(DiGiulio et al., 2015)
The absence of Lactobacillus spp. in the vagina during the first trimester of pregnancy may be associated with an increased risk of pregnancy loss in the second trimester.	(Nelson et al., 2007)
In this study, the authors describe that woman who suffered a miscarriage exhibited a predominance of potentially pathogenic bacteria, such as Lactobacillus iners and Gardnerella vaginalis, in their vaginal microbiome when compared to women who had successful pregnancies.	(Al-Memar et al., 2020)
Miscarriage and vaginal dysbiosis are related. The risk of miscarriage increases when vaginal *Lactobacillus* spp. levels fall during the first or second trimester of pregnancy.	(Nelson et al., 2015)
According to the authors of this study, having Lactobacillus iners in the vagina increases the chance of miscarriage.	(Shahid et al., 2022)
Gardnerella, Prevotella, Megastrobila, and Cyclospora, as well as an absence of Lactobacillus spp. in the vagina, could be causes of pregnancy loss.	(Xu et al., 2020)
In this study, the authors compared the women who had successful pregnancies, women who miscarried had lower levels of Lactobacillus crispatus and higher levels of harmful bacteria in their cervical mucus.	(Severgnini et al., 2022)
In this study, the authors discovered that compared to women without a history of miscarriage, women with a history of recurrent miscarriage had fewer Lactobacillus species in their vaginal microbiota.	(Xu et al., 2020)
High-risk pregnancies are linked to the abundance of Gardnella *vaginalis, Atopobium vaginae as well as Chlamydia trachomatis in the vaginal* which may cause miscarriage.	(Bretelle et al., 2015)
In this study, the authors found that women who have had a second-trimester miscarriage have a much greater frequency of vaginal dysbiosis when compared to those who have had repeated miscarriages.	(McPherson, 2016)
In this study, the authors showed that reduced *Lactobacillus* spp. associated with the growth of *Streptococcusas, Prevotella* and *Atopobium*, *well as RM.*	(Chang et al., 2020)
In this study, the authors reported that women had a recurrent pregnancy loss, she has a high level of *Gardnerella vaginalis* and other anaerobic bacteria in their vaginal microbiome.	(Peuranpaa et al., 2022)
In women with RM, *Lactobacillus* spp. is absent from the vagina.	(Kuon et al., 2017)
the authors of this study reported that women who experienced miscarriage had a higher abundance of pathogenic bacteria, such as *Ureaplasma* and *Mycoplasma*, in their amniotic fluid compared to women with successful pregnancies.	(Ahmadi et al., 2014)
In this study, the authors reported that women who experienced one miscarriage in the previous six months had been suffering from bacterial vaginosis.	(Isik et al., 2016)
In this study, the authors found that women had recurrent miscarriage, she had a lower level of *Lactobacillus species* and a higher abundance of pathogenic bacteria in their cervical mucus compared to women with successful pregnancies.	(Chen et al., 2017)

The risk of miscarriage has been associated with the vaginal microbiome through various mechanisms. For instance, first-trimester miscarriage is correlated with a decrease in *Lactobacillus* spp. abundance and an increase in the variety and richness of bacterial communities (Al-Memar et al., 2020). Also, *Group B Streptococcus* vaginal infection that causes exfoliation and subsequent bacterial ascension has been associated with higher rates of miscarriage (Vornhagen et al., 2018). *Atopobium, Prevotella, and Streptococcus* species were found in greater abundance in the recurrent miscarriage group than in the control group. Additionally, *Ureaplasma* levels were significantly higher in women who had a history of miscarriage (Zhang et al., 2019). On the other hand, a study compared the vaginal microbiome composition in women who had a miscarriage with those who delivered at term. The results showed that only 15% of women who gave birth at term and had no history of miscarriage had *Lactobacillus iners* as the predominant vaginal bacteria, compared to 50% of women with recurrent miscarriages (Nasioudis et al., 2017; Shahid et al., 2022).

The findings suggest that the prevalence of *Lactobacillus iners* in the vaginal microflora, and the presence of non-Lactobacillus species in vaginal preparations’ microscopy and chronic endometritis, are all independent predictors of early miscarriage. On the other hand, the dominance of *Lactobacillus crispatus* has been associated with a reduced risk of late miscarriage (Shahid et al., 2022). The correlation between low levels of *Lactobacilli* and pregnancy loss is noteworthy, as studies have demonstrated a link between decreased implantation and increased pregnancy loss in women undergoing *in vitro* fertilization who have low levels of *Lactobacillus* species (Singer et al., 2019). There was a clear correlation between greater log concentrations of *Leptotrichia/Sneathia* species or Megasphaera phylotype 1-like species in women and a decreased risk of miscarriage during early pregnancy (Nelson et al., 2015). Notably, previous studies examining the vaginal microbiota composition at the genus level in women with confirmed miscarriages has revealed a decrease in the number of Lactobacillus species during the first or second trimester of pregnancy (Al-Memar et al., 2020). Furthermore, it has been women diagnosed with dysbiosis during the first trimester have an increased risk of pregnancy loss in the second trimester, although the increase is not statistically significant. However, compared to those with a normal vaginal microbiota, among women with the most pronounced alterations in the vaginal microbiota, there was a twofold higher risk of pregnancy loss in the second trimester (Freitas et al., 2017).

Several studies have examined the association between recurrent miscarriage (RM) and *Lactobacillus* spp., as well as the growth of pathogenic bacteria in the vaginal microbiota. These studies focused on non-pregnant women with a history of three or more consecutive miscarriages (Llahi-Camp et al., 1996; Isik et al., 2016; Kuon et al., 2017; Zhang et al., 2019), using microscopic evaluation of Gram-stained vaginal smears. Research revealed that bacterial vaginosis (BV) was more common among women who experienced a single second-trimester miscarriage compared to those with RM (Llahi-Camp et al., 1996). Similarly, another study demonstrated a significant association between the presence of BV and a single miscarriage within the past six months, while no significant link was found between BV and recurrent miscarriages. Although some studies did not directly establish a connection between BV and RM, recent research utilizing 16S rRNA gene sequencing highlighted that women with RM exhibit higher genus richness in the vaginal microbiota compared to healthy women, with the identification of bacteria such as *Atopobium, Prevotella*, and *Streptococcus* (Zhang et al., 2019). Moreover, women with RM exhibited reduced levels of *Lactobacillus spp* (Kuon et al., 2017; Zhang et al., 2019; Fan et al., 2020). Almost 20% of patients with RM showed vaginal colonization by *G. vaginalis* and 15% by *Enterobacteriaceae*. Other studies also suggest that BV may contribute to chronic endometritis, which correlates with the occurrence of RM (Bardos et al., 2019; Kiecka et al., 2021). Toxoplasma gondii, an intracellular protozoan organism distinct from bacteria, holds the ability to infect a range of vertebrate species (Su et al., 2003). Research findings have indicated that toxoplasmosis can potentially lead to miscarriage as one of its complications (Hernández-Cortazar et al., 2016; Nayeri et al., 2020). Particularly during the early stages of pregnancy, prior to the critical phase of fetal organ development, this infection has been linked to a heightened risk of miscarriage (Kalantari et al., 2021). The exact mechanisms by which *Toxoplasma gondii* causes miscarriage are not fully understood, but it is believed to be related to the damage caused by the parasite to the developing fetus and the placenta (Kalantari et al., 2021).

*Chlamydia trachomatis* infection has been linked to miscarriage as it can induce faulty decidualization and chemokine release in human endometrial stromal cells (Giakoumelou et al., 2017). Other studies have reported inconsistent association between *Chlamydia trachomatis* infection and miscarriage (Baud et al., 2011; Giakoumelou et al., 2015; Rantsi et al., 2016).

One of the most prevalent viral infection of the reproductive system is human papillomavirus (HPV) (SYRJÄNEN et al., 1990; De Sanjosé et al., 2007). Notably, HPV infection is more prevalent in pregnant than in age-matched non-pregnant women (Fife et al., 1996; Castellsagué et al., 2009; Freitas et al., 2009). The prevalence of HPV infection also increases as pregnancy progresses (Schneider et al., 1987; Rando et al., 1989). HPV has been detected in 60% of spontaneous abortions and 20% of elective abortions, suggesting a potential involvement of HPV in the miscarriage’s pathogenesis (Hermonat et al., 1997). The proposed mechanism via which HPV impacts miscarriage is by inhibiting the placental trophoblast growth, decreases cell viability and induces cellular death (Chen et al., 2015). Recent studies have investigated the potential role of Polyomavirus BK virus infection on adverse pregnancy outcomes. However the results have mostly been inconclusive with no BK viral load detected in placenta from miscarriages of patients with unexplained villitis (infection of the placental villi associated with adverse pregnancy outcomes) (Cajaiba et al., 2011).

In a study involving frozen trophoblastic tissue samples from Greek women, two members of the herpes virus family, HSV-1 (HHV1) and HSV-2 (HHV-2), were identified in 43.5% of 95 cases with spontaneous pregnancy loss, in contrast to 16.7% of women undergoing elective abortion (Kapranos and Kotronias, 2009). While the authors did not differentiate between the types of HSV, their findings suggested a potential role of HSV in early miscarriages (Kapranos and Kotronias, 2009). In a more recent study, 500 pregnant women were tested for HSV-2 and 85 of them (17%) were seropositive (Kim et al., 2012). Most of the women in the cohort also tested positive for hepatitis B, Rubella, Varicella Zoster (HHV-3). Within the HSV-2 seropositive group, 38.8% had a history of miscarriage, compared to 29.6% in the control group (Kim et al., 2012). Collectively, bacterial, viral, and protozoan infectious agents have been linked to an elevated risk of miscarriage. However, further research is imperative to validate and substantiate the outcomes of these studies.

## 5 Increased levels of proinflammatory cytokines are associated with higher risk of pregnancy loss:

The vaginal nature is equipped by various immune cells and receptors that can identify and react to microorganisms (Wira et al., 2005). However, changes in the microbiota and its relations with the immune system might cause pregnancy complications, including loss of pregnancy. According to Villa et al. discovered that the vaginal squamous epithelial cells and the columnar cells in the upper female genitalia can detect both commensal and pathogenic bacteria, by activating pattern recognition receptors such as nucleotide-binding oligomerization domain (NOD), Toll-like receptors (TLRs), dectin-1 receptor (Buchta, 2018). Moreover, women who frequently have recurrent spontaneous miscarriage (RSM) are prone to producing more embryotoxic Th1 cytokines in response to human trophoblast antigens in their peripheral blood lymphocytes.

**Figure 3:**
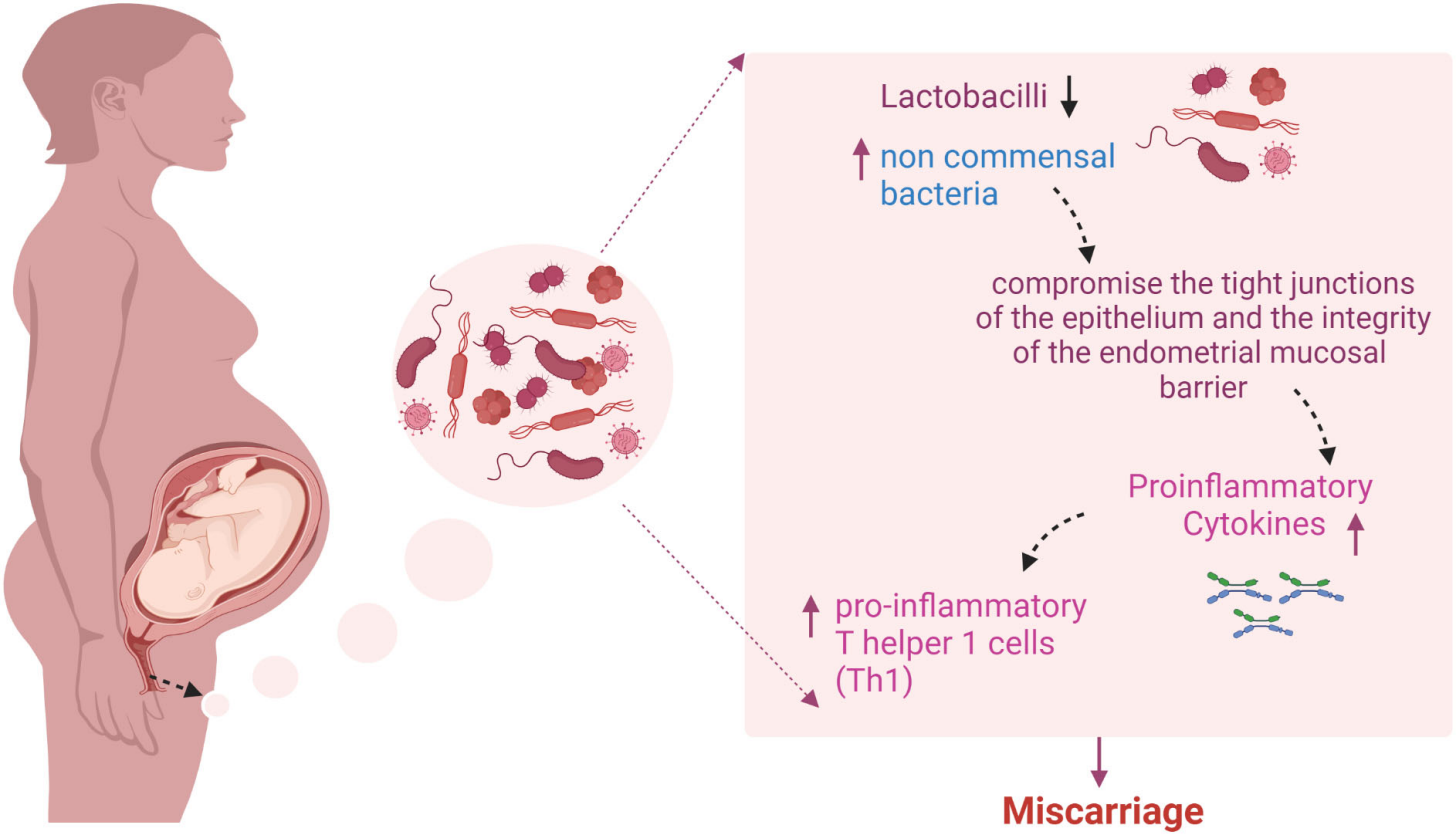
Predominance of non-commensal bacteria and miscarriage: The overabundance of non-commensal bacteria has the potential to disrupt the tight connections of the epithelium and compromise the integrity of the endometrial mucosal barrier. If the host’s defense mechanisms become compromised, pathogens may enter the endometrial stroma and activate an immune response in antigen-presenting cells and other immune cells that express Pattern-recognition receptors (PRRs). Bacterial products that are ingested or infections that penetrate and rupture the mucosal barrier may activate T cells. An imbalance in cytokine production may favor the production of pro-inflammatory T helper 1 cells (Th1) that generate tumor necrosis factor (TNF), interferon (IFN), and interleukin-2 (IL-2). The figure was created with BioRender.com.

In 2020, Al-Nasirya et al. suggested a few possible routes that may be involved in how bacteria affect the implantation process (**Figure 3**). One pathway is the predominance of non-commensal bacteria, which can compromise the endometrial mucosal barrier’s integrity and the epithelium’s tight connections. In case the host defense mechanisms fail, pathogens can enter the endometrial stroma and activate an immune response in antigen-presenting cells (APCs) and other immune cells that express pattern recognition receptors. Bacterial products or infections that penetrate and rupture the mucosal barrier can activate T cells. However, an imbalance in the production of cytokines may encourage the production of pro-inflammatory T helper 1 cells (Th1) may be favored because of an imbalance in cytokine production.

## 6 The mechanisms of miscarriage relating to vaginal microbiome:

The exact mechanisms linking changes in the vaginal microbiome to miscarriage are not fully understood. However, several potential mechanisms have been proposed based on research findings and hypotheses, encompassing factors such as inflammation, infection, immunological responses, microbial translocation, hormonal influence, and the ascent of pathogens (Giakoumelou et al., 2016; El Hachem et al., 2017).

Changes in the vaginal microbiome can result in an excess of certain pathogenic bacteria or a reduction in beneficial bacteria, for example decreased abundance of vaginal *Lactobacillus* spp. and increased bacterial community diversity and richness, ultimately resulting in a state of dysbiosis. This dysbiosis can cause an inflammatory response inside the vaginal tissues, resulting in inflammation of the cervix and its surrounding structures (Chee et al., 2020; Chen et al., 2021; Lehtoranta et al., 2022; Holdcroft et al., 2023). Furthermore, alterations in the vaginal microbiota have been linked to illnesses such as bacterial vaginosis (BV), which might be associated with miscarriage (Lewis et al., 2017).

The vaginal microbiome plays a role in regulating the local immune response within the female reproductive system (Adapen et al., 2022). Changes in the vaginal microbiome could disrupt the immunological balance, causing immune dysregulation and increasing the chance of miscarriage: for example Infection of human endometrial stromal cells with *Chlamydia trachomatis* has been associated with defective decidualization and impaired chemokine production, which can lead to miscarriage (Bardos et al., 2019; Grewal et al., 2022). Similarly, dysbiosis in the vaginal microbiome could compromise the integrity of cervical tissues or cause cervix inflammation, resulting in cervical insufficiency (Mitra et al., 2016; Ma et al., 2023). This disorder causes early dilation of the cervix, which can lead to miscarriage. According to certain studies, harmful bacteria from the vaginal microbiome may migrate to the uterine cavity or even reach the placenta during pregnancy. This movement may cause an inflammatory response within the placental tissues, thereby impacting fetal development and increasing the likelihood of miscarriage (Bardos et al., 2019; Wang et al., 2021).

## 7 Recommendations to prevent early miscarriage:

It’s important to note that some early miscarriages are caused by genetic abnormalities or other unknown causes. However, there are recommendations that can promote a healthy pregnancy and decrease the risk of miscarriage:

- Seek Early prenatal care: Schedule an appointment with your healthcare provider for early prenatal care as soon as you become pregnant. This will help identify any health issues or risks that may lead to an early miscarriage.
- Actively treat any vaginal infection that might change the normal vaginal environment and ecosystem.
- Avoid use of vaginal washes that might change the normal vaginal environment and ecosystem.
- Avoid smoking, alcohol, and illicit drugs: Smoking, alcohol, and drug all raise the chance of miscarriage.
- Maintain a healthy diet: Consuming a well-balanced diet that includes a variety of fruits, vegetables, healthy grains, and lean proteins may help reduce the risk of miscarriage.
- Stay active: Physical activity can assist in maintaining general health and lower the chance of difficulties during pregnancy. Before becoming pregnant, discuss safe and appropriate exercise options with your healthcare provider.
- Manage stress: Since stress during pregnancy increases the chance of miscarriage, it’s critical to discover techniques to control stress. This may involve engaging in relaxation exercises such as yoga or meditation or seeking help from a counselor or therapist.
- Avoid being exposed to dangerous substances: Exposure to several chemicals, pollutants, and radiation might raise your risk of miscarriage. As much as possible, stay away from these things, and if you have any questions, consult your doctor.

To prevent miscarriage, it is crucial to have regular prenatal care and avoid any potential risk factors. Regularly scheduling prenatal appointments with a healthcare provider is of paramount importance. Timely and consistent check-ups play a pivotal role in monitoring the progression of pregnancy and promptly identifying any potential issues. Maintaining a healthy lifestyle involves consuming nutritious food, staying hydrated, and refraining from harmful substances like alcohol, tobacco, and drugs. Furthermore, it is advisable to undergo early prenatal screenings and examinations, encompassing blood tests, ultrasounds, and genetic testing, to proactively detect any latent complications. A cornerstone of effective prenatal care involves heeding the guidance of medical professionals and taking prescribed prenatal vitamins, notably including essential nutrients like Folic acid.

While our understanding of the role of vaginal microbiome in miscarriage continues to grow, further studies are required to completely understand the mechanisms and establish a causal link. Nevertheless, continued research has the potential to shed light on the causes, prevention, and management of miscarriages. It may also result in the development of focused therapies, such the probiotics outlined in the subsequent section.

Invasive methods like chorion villus sampling and amniocentesis are likely to be replaced in the near future by diagnostic tests on fetal genetic material collected from maternal plasma for the prenatal diagnosis of fetal genetic abnormalities (Chiu and Lo, 2013). As early as week seven of pregnancy and with the help of next-generation sequencing methods, fetal aneuploidies can already be detected from cell-free fetal DNA recovered from the mother’s blood (Kitzman et al., 2012). However, there is still a need for further research to understand the relationship between the vaginal microbiota and miscarriage and to develop effective methods to prevent or treat dysbiosis in pregnant women.

## 8 Microbiota based interventional strategies to prevent and treat miscarriage

The composition of the vaginal microbiota can be influenced by various factors, including antibiotics, pre and probiotics, and other variables (Macklaim et al., 2015). Consequently, these parameters may serve as intervention tools to modify the vaginal microbiome and achieve the desired health outcomes. Probiotics encompass living or active microorganisms intended to enhance health when consumed orally or applied topically (Hill et al., 2014). On the other hand, prebiotics are foods that serve as a substrate for healthy human microbiota (usually high-fiber meals) (Hill et al., 2014). As previously indicated, a reduction in *Lactobacillus* spp. within the vaginal microbiota has been observed in women who have experienced confirmed miscarriages (Shahid et al., 2022). While studies examining the utilization of prebiotics and probiotics for miscarriage prevention are relatively limited, the notion of employing *lactobacilli* species as probiotics or using prebiotics to foster the growth of protective organisms emerged in the mid-1980s. *L. rhamnosus GR-1* and *L. reuteri RC-14* were among the first probiotics used to improve vaginal health (Reid et al., 1987). Pilot human studies revealed that the GR-1 strain was effective at inhibiting gram-negative pathogens, can be kept in the vagina and help delay onset of infection (Bruce and Reid, 1988; Bruce et al., 1992). On the other hand, *Lactobacillus RC-14* showed a superior capacity to create biosurfactant materials that considerably hindered gram-positive coccal adhesion (Velraeds et al., 1996; Heinemann et al., 2000). According to one study, *L. rhamnosus* GR-1 and *L. reuteri* RC-14 delivered via capsule can cure BV (Anukam et al., 2006). The oral consumption of probiotics has been substantiated to decrease the presence of irregular microbiota, such as *Gardnerella* and *Atopobium*, while concurrently augmenting the abundance of the *Lactobacillus* genus (Bohbot and Cardot, 2012; Bhandari and Prabha, 2015; López-Moreno and Aguilera, 2021). *Lactobacilli* have been administered orally with milk and yogurt; both of which may have a prebiotic effect to safeguard the organisms through the GI tract and lower the risk of urogenital infection (Beereport et al., 2009). Additionally, it has been demonstrated that *consuming L. rhamnosus GR-1* from yogurt improves intestinal and immune functions in people with HIV (Irvine et al., 2010). Additionally, it has been demonstrated that using probiotics in addition to antibiotics or antifungals not only to mitigate the adverse effects of antibiotics/antifungals but also to improve the efficacy of treatment for BV and Vulvovaginal Candidiasis (VVC) (Anukam et al., 2006). Other studies have demonstrated the beneficial effects of mixed *Lactobacillus-*based probiotics in reducing recurrence rates and symptoms including vaginal discharge and itching (Anukam et al., 2006; Laue et al., 2018; Russo et al., 2019; Reznichenko et al., 2020).

While antibiotics are not primarily indicated for miscarriage prevention, they are often administered when surgical procedures are necessitated to remove uterine tissue following pregnancy loss (Verma and Crespo, 2020). Infection following miscarriage can have long-term effects such as pelvic scarring, a rise in ectopic pregnancy rates, and infertility (Melese et al., 2017). Infections can also cause significant sickness and even death. When performing certain surgical procedures, such as vacuum aspiration for abortion, antibiotics are given as a preventative measure to lessen postoperative infection (Ahmed et al., 2022). Prophylactic antibiotics are shown to prevent pelvic infection when given during surgical abortion, according to a Cochrane analysis of 19 randomized studies (Low et al., 2012). Additionally, studies have shown that prophylactic antibiotics are prescribed for uterine evacuation procedures to treat miscarriage in almost 50% of women in some countries (Fawcus et al., 1997), and that prophylactic antibiotics may improve outcomes (Melese et al., 2017). Therefore, it seems that there are some benefits to the use of prophylactic antibiotics for preventing infections for women undergoing or undergone miscarriage surgery. Other drugs such as low dose aspirin and metformin have also shown promise to reduce the risk of miscarriage in women with autoimmune disorders, certain blood clotting disorders and polycystic ovary syndrome (Jakubowicz et al., 2002; Naimi et al., 2021). In conclusion considering the importance of the “healthy” vaginal microbiota in preventing miscarriages, the future will see an expanded use of probiotics, prebiotics and other drugs for vaginal health (National Centre for Biotechnology Information, ).

## Conclusion

In conclusion, although the causes of miscarriage are often unknown, research suggests that the microbiota in the vaginal could contribute to an increase in miscarriages. Understanding this connection could help prevent certain cases and improve mental health by promoting acceptance. However, additional research is required to fully comprehend the link between the vaginal microbiome and miscarriage, and to develop effective methods for treating or preventing dysbiosis in pregnant women. By gaining a better understanding of the female genital tract environment and identifying new biomarkers, we can improve gynecological evaluations and develop personalized therapies for women. In the end, this could help in improved diagnosis procedures and pregnancy outcomes.

## Author contributions

MS and SA wrote the first draft. PS helped addressing reviews comments. OO and SK reviewed and finalized the manuscript. All authors contributed to the article and approved the submitted version.
